# Triploid cultivars of *Cymbidium* act as a bridge in the formation of polyploid plants

**DOI:** 10.3389/fpls.2022.1029915

**Published:** 2022-10-11

**Authors:** Man-Man Li, Qing-Lian Su, Jun-Rui Zu, Li Xie, Qian Wei, He-Rong Guo, Jianjun Chen, Rui-Zhen Zeng, Zhi-Sheng Zhang

**Affiliations:** ^1^ Guangdong Province Key Laboratory of Plant Molecular Breeding, College of Forestry and Landscape Architecture, South China Agricultural University, Guangzhou, China; ^2^ Guangzhou Flower Research Center, Guangzhou, China; ^3^ Mid-Florida Research and Education Center, Environmental Horticulture Department, Institute of Food and Agricultural Sciences, University of Florida, Apopka, FL, United States

**Keywords:** cross compatibility, *Cymbidium*, polyploidy, triploid bridge, unreduced gamete

## Abstract

Triploid is considered a reproductive barrier and also a bridge in the formation of polyploids. However, few reports are available in *Cymbidium*. In this study, diploid ‘Xiaofeng’, sexual triploid ‘Yuchan’ and ‘Huanghe’ of *Cymbidium* were used to evaluate hybridization compatibility of the triploids. Results showed that the sexual triploids were fertile whether they were used as male or female parents. ‘Yuchan’ produced male gametes of 1*x*, 1*x*~2*x*, 2*x*, 2*x*~3*x*, and 3*x* at frequencies of 8.89%, 77.78%, 6.67%, 3.33%, and 3.33%, respectively; while ‘Huanghe’ produced 3.33% 1*x*, 80.00% 1*x*~2*x*, 8.89% 2*x*, 5.56% 2*x*~3*x*, and 2.22% 3*x* male gametes. The cross of ‘Xiaofeng’ with ‘Yuchan’ produced progenies with a wide range of ploidy levels, including one diploid, 34 2×~3× aneuploids, 12 triploids, and one tetraploid, indicating that male gametes produced by sexual triploid were fertile and could be transmitted and fused with egg cells. On the other hand, 10 progenies obtained from the cross of ‘Yuchan’ × ‘Xiaofeng’ were all aneuploids. The cross of ‘Yuchan’ with ‘Huanghe’ produced 40 progenies including three 2×~3× aneuploids, nine 3×~4× aneuploids, 21 tetraploids, six 4×~5× aneuploids, and one pentaploid, suggesting that 2*x* gametes, instead of the unreduced ones played a more important role in the formation of tetraploids. The survival rates of the hybrids were all above 80.00%, with the tetraploids at 96.67%. Cytological analysis revealed that during meiosis of sexual polyploids, two chromosome sets of the 2*n* gamete were inclined to enter into the same daughter cell, resulting in the production of 2*x* gametes. Our results indicate that the triploid cymbidiums are not reproductive barrier but serve as a bridge in the formation of polyploid plants.

## Introduction

Polyploidy plays a major role in the evolution and diversification of plants (Thompson and Lumaret, 1992; Bretagnolle and Thompson, 1995; Kovalsky et al., 2018). In natural populations, polyploidy is formed by several different routes (Ramsey and Schemske, 1998). Among them, sexual polyploidy through unreduced gametes (2*n* gametes) is considered to be the main pathway (Bretagnolle and Thompson, 1995; Ramsey and Schemske, 1998; Xie et al., 2022). The union of reduced and unreduced gametes produces triploids, and the combination of two unreduced gametes forms tetraploids (Bretagnolle and Thompson, 1995; Husband, 2004). Owing to the limited chance in the fertilization between simultaneously formed unreduced male and female gametes, triploids are usually considered as the intermediate stage in the formation of stable tetraploids, and this pathway of tetraploid formation is known as the ‘triploid bridge’ (Ramsey and Schemske, 1998; Yamauchi et al., 2004; Jike et al., 2020).

Triploids play an important role in polyploidy dynamics of natural populations (Husband, 2004). For example, 1% tetraploid progeny were obtained by backcrossing a spontaneous triploid clone of *Populus tremula* with a diploid (Bergstrom, 1940). Henry et al. (2005) reported that triploid *Arabidopsis thaliana* plants were fertile and could lead to the formation of tetraploids because they act as bridges between euploid types. Schinkel et al. (2017) revealed that a female triploid produced through unreduced egg cells was the major cause of polyploidization in *Ranunculus kuepferi*. In the cross of 2× × 3×[2x] of *Chamerion angustifolium*, 65% progeny were triploids and 16% were tetraploids, while 45% triploid progeny and 35% tetraploid progeny were produced in the cross of 2× × 3×[4x] (Burton and Husband, 2001). Using triploid as parents, tetraploids and/or pentaploids were produced through the cross of triploid × diploid in *Hieracium echioides* (Peckert and Chrtek, 2006), *Phalaenopsis* (Zhou et al., 2009), *Tulipa* (Marasek-Ciolakowska et al., 2014), and *Phegopteris* (Nakato and Masuyama, 2021). Hexaploids were obtained from the selfing progeny of triploid *Phegopteris decursivepinnata* (Nakato and Masuyama, 2021).

A challenge to the formation of higher polyploidy *via* the ‘triploid bridge’ pathway is the occurrence in aneuploid gametes which can substantially reduce fertility (Ramsey and Schemske, 1998; Husband, 2004; Duszynska et al., 2019). However, increasing evidence has suggested that triploids can produce functional euploid (n = *x*, 2*x* or 3*x*) and aneuploid male gametes in some species (Ramsey and Schemske, 1998; Diao et al., 2009; Czarnecki et al., 2014; Kovalsky et al., 2018). Further studies show that pollen fertility (or percentage of viable pollen) in triploid plants varies among species and cultivars (Jones and Reed, 2007; Farco and Dematteis, 2014; Zhang et al., 2017; Alexander, 2020). Pollen fertilities in some triploid *Turnera sidoides* were found to be greater than 60% (Kovalsky et al., 2018; Alexander, 2020) and reach up to 80% in triploid *Hydrangea macrophylla* (Jones and Reed, 2007) and even 90% in triploid white poplar plants (Wang et al., 2010).

Another obstacle restricting the role of triploids in polyploid evolution is ‘triploid block’. Triploid block, which prevents interploidy hybridization, is characterized by abnormal endosperm development and seed collapse (Johnston et al., 1980; Erilova et al., 2009; Koehler et al., 2010; Schinkel et al., 2017; Huc et al., 2022). It is well known that the endosperm may develop abnormally in interploidy-intraspecific crosses if the maternal and paternal genome deviates from 2:1 ratio (Johnston et al., 1980; Vinkenoog et al., 2003; Haig, 2013). However, some deviations from this ratio are found to be allowable in certain species as viable seeds were produced in *Zea mays* (Lin, 1984), *Solanum tuberosum* (Ehlenfeldt and Ortiz, 1995), *Arabidopsis thaliana* (Scott et al., 1998), and *Peperomia* (Friedman et al., 2008).

Orchids are plants belonging to the family Orchidaceae that are prized by their ornamental and medicinal value. There are 27,801 recognized species that are globally distributed with the exception of Antarctica (Vilcherrez-Atoche et al., 2022). Orchids have been used as models for studying plant evolutionary processes and adaptability to different environmental conditions. Polyploidy plays an important role during the evolution of orchids as sequence analyses showed that whole-genome duplication (WGD) occurred widely in orchids, including *Apostasia shenzhenica* (Zhang et al., 2017; Zhang et al., 2021), *Cymbidium ensifolium* (Ai et al., 2021), *Dendrobium chrysotoxum* (Zhang et al., 2021), and *Phalaenopsis* (Cai et al., 2015). A recent study showed that triploids clustered in an intermediate position between diploids and tetraploids in *Zygopetalum mackayi* (Moura et al., 2020). In the *Nigritella nigra* group, nuclear and plastid marker analysis showed that tetraploid *N. nigra* subsp. *austriacais* somewhat differentiated from the triploid subsp. *Nigra* at nuclear as well as plastid loci. The fusion of an unreduced egg cell from subsp. *Nigra* with a haploid microgamete from *Gymnadenia conopsea* gave rise to *Gymnigritella runei* (Hedren et al., 2018). In *Phalaenopsis*, diploids, triploids, pentaploids, and aneuploids were produced from the crosses of diploid × triploid or triploid × diploid. Triploids, tetraploids, octoploids, and aneuploids were identified in triploid × tetraploid crosses, while no hybrids were obtained from the cross of triploid × triploid (Zhou et al., 2009). Nevertheless, it is generally acknowledged that polyploids can be formed *via* polyspermy, unreduced gamete, hybridization and endopolyploidy in orchids (Okamoto et al., 2017; Vilcherrez-Atoche et al., 2022); but it is still unclear how each of these pathways contributes to the polyploidization in orchids.

*Cymbidium* Sw. is one of the most important orchids consisting of 74 species that are epiphytic, lithophytic, terrestrial or sometimes rarely leafless saprophytic (Ning et al., 2018; Thakur and Dutt, 2021). Among the terrestrial species, *C. sinense*, *C. ensifolium, C. goeringi*, and *C. kanran* are the most popular flowering ornamental plants and widely cultivated for their beauty and fragrance (Huang et al., 2012). Our previous research identified 2*n* gamete occurrence in cultivated cymbidiums (Zeng et al., 2020). Hybridization among selected cultivars or species produced five triploid and two tetraploid progenies. Two of five triploids were propagated through *in vitro* culture and evaluated in shaded greenhouse for their aesthetic value. Results showed that they had improved ornamental traits displayed by rounder flowers with wider sepal, petals, and lips compared to the diploids. The occurrence of more triploids than tetraploids was intriguing. Since orchids do not have endosperm, triploid block due to the endosperm balance could not be great concern. Besides, triploid plants can be easily propagated through *in vitro* culture (Zeng et al., 2020). The higher frequency in triploid occurrence, the improved ornamental traits, and little concern over the triploid block prompted us to further analyze 2*n* gamete occurrence in cymbidiums and the implication of triploids as a bridge in the formation of polyploid plants.

The objectives of this study were to examine microsporogenesis and microgametogenesis behaviors of two sexual triploids, determine their pollen type and fertility, evaluate their crossability with either diploids or triploids, and analyze ploidy levels and the survival rates of their progenies. Results showed that the union of 2*x* gametes, which were derived from the unreduced gamete, was probably the key pathway for the formation of tetraploids through ‘triploid bridge’. Our studies with cymbidium demonstrated the importance of triploids in the formation of polyploid plants.

## Materials and methods

### Plant materials

A total of seven cultivars were used in this study (**Supplementary Figure S1**). Two of them, ‘Yuchan’ and ‘Huanghe’, were sexual triploids. The remaining ‘Xiaofeng’, ‘11-65-1004’, ‘13-44-5’, ‘12L-2018-2’, and ‘Gongfenjiaren’ were diploids. Plants were grown in a shaded greenhouse under a light intensity of 120 µmol·m^-2^·s^-1^, temperature ranging from 15°C to 30°C, and relative humidity varying from 70 to 80% at the Experimental Farm of South China Agricultural University, Guangzhou, China. At anthesis, the following studies were performed with selected cultivars.

### Cytological observations of microsporogenesis and microgametogenesis

Microsporogenesis and microgametogenesis were observed using the method described by Zhu et al. (2014). The pollinia of ‘Yuchan’, ‘Huanghe’, and ‘Xiaofeng’ at different formation and developmental stages were collected and fixed at 4°C for 12-24 hours in fresh prepared Carnoy’s solution. They were then transferred to 70% ethanol and stored at 4°C. The fixed pollinia were placed on a slide; after surface dried with filter paper, two drops of improved carbolic acid fuchsin or 4,6-diamidino-2-phenylindole (DAPI) [2 µg.ml^−1^ DAPI, 1% Triton X-100 (v/v), and 1% sucrose (w/v)] staining solution were added, they were crashed with a forceps, and stained in the dark at room temperature for 5 min. A cover glass was applied and squeezed with pencil eraser, the slide was observed and photographed under either light or UV illumination with ZEISS microscope. For observation of each microsporogensis stage per hybrid progeny, at least nine slides were observed where three slides and 100 microsporocytes as a replicate. There were three replications for each hybrid progeny. For examining each microgametogensis stage of the hybrid progenies, at least ninety pollens were observed with thirty pollens as a replicate. The observations also had three replications. The percentage of meiosis abnormalities and each male gamete type were calculated as follows: (1) the percentage of meiosis abnormalities = (the number of abnormal microspore mother cell in a replicate/100) × 100% and (2) the percentage of each male gamete type = (the number of certain male gamete type in a replicate/30) × 100%.

For the calculation of dyad and triad occurrence, ten vision fields were photographed at 40× magnitude for each slide, one slide was regarded as a replicate, and each material was replicated three times. The frequencies of dyad and triad incidence were calculated according to the formula: F_Dy_(%) = (Number of dyads/total microspore count observed)) ×100; F_Tr_(%) = (Number of triads/total spore count observed) ×100.

### Pollen viability determination

Pollinia of ‘Yuchan’ or ‘Xiaofeng’ were collected from the flowers that had opened for one day and placed on a slide. After two or three drops of 0.05% of 2, 3, 5-triphenyltetrazolium chloride solution were added, the pollinia were crashed with a forceps and kept in the dark at room temperature for 2-3 h. A coverslip was applied, and the slide was observed and photographed under photomicroscope (OlympusIX71, Japan). Pollen grains with red color were regarded as viable. About 1,000 pollens were counted per slide, one slide was regarded as a replicate, and three slides per cultivar were observed. The pollen fertility was calculated according to the formula: The pollen fertility (%) = (the number of stained pollen grains/total number of pollen grains observed) × 100.

### Hybridization, seed germination, seedling production and transplanting

Methods of hybridization, seed germination, seedling production and transplanting were described previously (Zeng et al., 2020). A total of 11 pairs of hybridization were made using the seven cultivars. The numbers of pollinated flowers and capsules produced from the pollinations were recorded, and fruit setting rates were calculated. Seeds harvested from the cross of ‘Yuchan’ × ‘Xiaofeng’, ‘Xiaofeng’ × ‘Yuchan’, and ‘Yuchan’ × ‘Huanghe’ were germinated *in vitro*. The protocorm like body or rhizome obtained from a seed was propagated, and test-tube seedlings were produced (Zeng et al., 2020). After the seedlings reached about 5 cm in height, they were used for identification of ploidy levels. When the seedlings were about 8 cm in height, they were removed from test tubes, rinsed with tap water, briefly air dried, and transplanted into small black plastic planting bags (100 mL). Each bag was filled with a substrate comprised of small pine bark (1 cm in length) and peat in a 3:1 ratio based on volume, one seedling per bag. Potted seedlings were grown in the aforementioned shaded greenhouse and fertigated with a Hyponex (N–P_2_O_5_–K_2_O; 20–20–20) solution every 10 d. After 6 months of growth, they were transplanted into 2.6 L bags filled with the pine bark and granite substrate and grown in another shaded greenhouse under a light intensity of 300-400 µmol·m^-2^·s^-1^. A slow-release fertilizer (N–P_2_O_5_–K_2_O; 20–20–20) was applied to each bag at 3-4 g each in March and September, respectively. Meanwhile, a solution containing 0.1% KH_2_PO_4_ (w/v) was sprayed monthly during growing season. Initially, a total of 90 seedlings from each hybrid were transplanted, and they were arranged as a randomized complete block design with three replicates. After 10 months of transplanting, the number of surviving seedlings were recorded, and the survival rate was calculated according to the formula: The survival rate (%) = (the number of seedlings survived in a replicate/30) × 100.

### Flow cytometry analysis

The ploidy level of hybrid progenies was measured by flow cytometry (Cui et al., 2009; Zeng et al., 2020). For each individual, young leaves, about 0.5 cm^2^, were placed in a one-off culture dish. After adding 0.4 mL of PartecHR-A extract, the leaves were chopped quickly with a blade, following by adding 1.6 mL of Partec HR-B (DAPI, 4,6-diamidino-2-phenylindole) solution as DNA staining agent. The mixture samples were filtered through 30 μm Partec Celltrics microporous membrane, stained in darkness for 5 min and analyzed by Partec flow cytometer using CyView8.5 software (PartecGmb H, Munster, Germany). The DNA histograms of nuclei from each sample were produced.

### Chromosome counts

In order to further verify ploidy level of the hybrid progenies, the number of chromosomes in root tip cells was accounted by squash method (Zhou et al., 2009). The root tips with a length of 2~3 mm was cut from the newly formed root of *in vitro* grown seedlings and fixed in Carnoy’s solution, which was consisted of 95% ethanol and glacial acetic acid in a 3:1 ratio based on volume, at 4°C for 12~24 h. The fixed material was washed with distilled water for 2~3 times, and then acidulated with 1 mL concentration of 1 mol·L^-1^ HCl in a constant temperature water bath at 60°C for about 8 minutes. The dissociated root tips were immersed in distilled water for 30 min, then stained with improved carbolic acid fuchsin staining solution, crushed with tweezers. The debris was discarded, and the sample was covered with a coverslip and observed at 100 × magnification using a photomicroscope (OlympusIX71, Japan). A digital camera system (Nikon) was used for photography. For each plantlet, at least 20 cells were observed. If more than 90% of the cells had a constant chromosome number, the chromosome number of the seedlings was confirmed. As diploid *Cymbidium* has somatic chromosome numbers of 40, we defined that chromosome numbers of 41-59, 61-79, and 81-99 were aneuploid of 2×~3 ×, 3×~4×, and 4×~5×, respectively.

### Statistical analysis

All data were subjected to analysis of variance using Microsoft Office Excel 2019 and SPSS 26.0 (IBM Corporation, Somers, NY). When significance occurred, means were separated by Duncan’s multiple range test at *P*< 0.05 level.

## Results

### Triploid pollen viability and hybridization compatibility

The intention of making the 11 crosses (**Table 1**) was to assess the hybridization compatibility of two sexual triploids ‘Yuchan’ and ‘Huanghe’. As a result, capsules were obtained in all 11 cross combinations, and all capsules obtained had seeds. The seeds collected from the crosses of ‘Yuchan’ × ‘Xiaofeng’, ‘Xiaofeng’ × ‘Yuchan’, and ‘Yuchan’ × ‘Huanghe’ were geminated normally *in vitro*, and the seedlings grew vigorously (**Supplementary Figure S2**). These results suggested that sexual triploid *Cymbidium* had high hybridization compatibility and could be used as male or female parent for hybridization.

**Table 1:** Fruit setting rates of 11 crosses made by using sexual triploid cultivars as one ortwo parents in *Cymbidium*.

Cross combination (♀ × ♂)	Year	No. of flowers Pollinated	No. of capsules obtained
‘Yuchan’ (3×) × ‘Xiaofeng’ (2×)	2018	1	1
‘Xiaofeng’ (2×) × ‘Yuchan’ (3×)	2018	2	2
‘Yuchan’ (3×) × ‘Huanghe’ (3×)	2018	1	1
‘Xiaofeng’ (2×) × ‘Yuchan’ (3×)	2019	1	1
‘11-65-1004’ (2×) × ‘Yuchan’ (3×)	2019	1	1
‘Yuchan’ (3×) × ‘13-44-5’ (2×)	2020	1	1
‘Yuchan’ (3×) × ‘12L-2018-2’ (2×)	2020	1	1
‘Yuchan’ (3×) × ‘Gongfenjiaren’ (2×)	2021	7	7
‘Gongfenjiaren’ (2×) × ‘Yuchan’ (3×)	2021	7	7
‘Yuchan’ (3×) × ‘Yuchan’ (3×)	2021	2	2
‘Huanghe’ (3×) × ‘Yuchan’ (3×)	2021	2	1

### Ploidy levels of hybrid progenies

**Figure 1:**
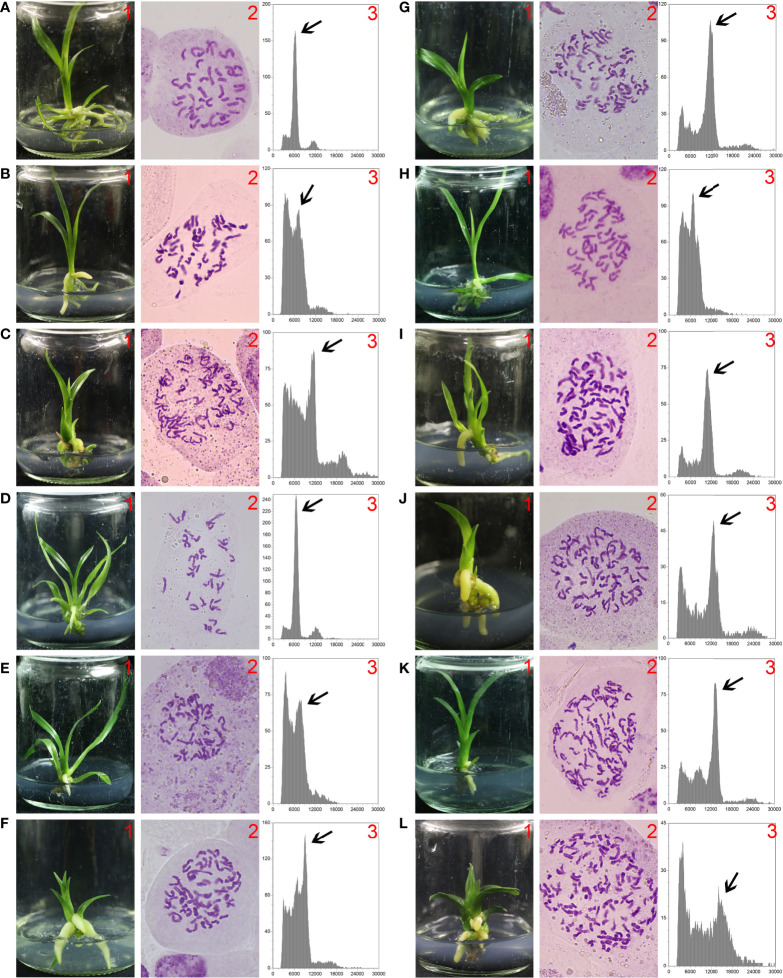
Ploidy identification of hybrid progenies. 1. *In vitro* cultured seedlings; 2. Chromosome numbers in a root tip cell; 3. Flow cytometry histogram of leaf tissue (arrow represents plant ploidy). **(A)** ‘Xiaofeng’ (diploid, 2*n* = 2*×* = 40). **(B, C)** Hybrid progenies of ‘Yuchan’ × ‘Xiaofeng’: **(B)** ‘18-21-8’ (aneuploidy, 2*n* = 50), **(C)** ‘18-21-10’ (aneuploidy, 2*n* = 75). **(D–G)** Hybrid progenies of ‘Xiaofeng’ × ‘Yuchan’ where **(D)** ‘18-50-1’ (diploid, 2*n* = 2*×* = 40), **(E)** ‘18-50-86’ (aneuploidy, 2*n* = 48), **(F)** ‘18-50-125’ (triploid, 2*n* = 3*×* = 60), and **(G)** ‘18-50-140’ (tetraploid, 2*n* = 4*×* = 80). **(H–L)** Hybrid progenies of ‘Yuchan’ × ‘Huanghe’ where **(H)** ‘18-24-50’ (aneuploidy, 2*n* = 56), **(I)** ‘18-24-33’ (aneuploidy, 2*n* = 72), **(J)** ‘18-24-15’ (tetraploid, 2*n* = 4*×* = 80), **(K)** ‘18-24-1’ (aneuploidy, 2*n* = 93), and **(L)** ‘18-24-172’ (pentaploid, 2*n* = 5*×* = 100).

The ploidy levels of progenies derived from the crosses of ‘Yuchan’ × ‘Xiaofeng’ and ‘Xiaofeng’ × ‘Yuchan’ were analyzed by DNA flow cytometry and root tip chromosome count (**Figure 1**). Results showed that among 10 identified progenies of ‘Yuchan’ × ‘Xiaofeng’, nine were aneuploids of 2×~3×, and one was the aneuploid of 3×~4×. In the reciprocal cross of ‘Xiaofeng’ × ‘Yuchan’, the percentages of aneuploid of 2×~3×, diploid (2×), triploid (3×), and tetraploid (4×) in the hybrid progenies were 70.8%, 2.1%, 25.0%, and 2.1%, respectively (**Table 2**).

**Table 2:** Ploidy level of hybrid progenies resulted from crosses with sexual triploid *Cymbidium*.

Cross combinations(♀ × ♂)	Total no. of plantlets evaluated	No. of plantlets with specified ploidy level						
		2×	2×~3×	3×	3×~4×	4×	4×~5×	5×
‘Yuchan’ (3×) × ‘Xiaofeng’ (2×)	10	0	9	0	1	0	0	0
‘Xiaofeng’ (2×) × ‘Yuchan’ (3×)	48	1	34	12	0	1	0	0
‘Yuchan’ (3×) × ‘Huanghe’ (3×)	40	0	3	0	9	21	6	1

The ploidy levels of hybrid progenies from the cross of ‘Yuchan’× ‘Huanghe’ were shown in **Figure 1** and **Table 2**. They were aneuploids of 2×~3×, 3×~4×, and 4×~5×; tetraploid, and pentaploid. The proportion of tetraploid was the highest, accounting for 52.5%, followed by aneuploids of 2×~3×, 3×~4×, and 4×~5× with proportions of 7.5%, 22.5%, and 15.0%, respectively. The proportion of pentaploid was the lowest (2.5%). The occurrence in higher proportion of tetraploid in the triploid × triploid cross suggested that the cross between triploids was probably a main avenue for producing polyploids with higher ploidy levels in the natural population.

### Types of male gamete and pollen fertility

**Figure 2:**
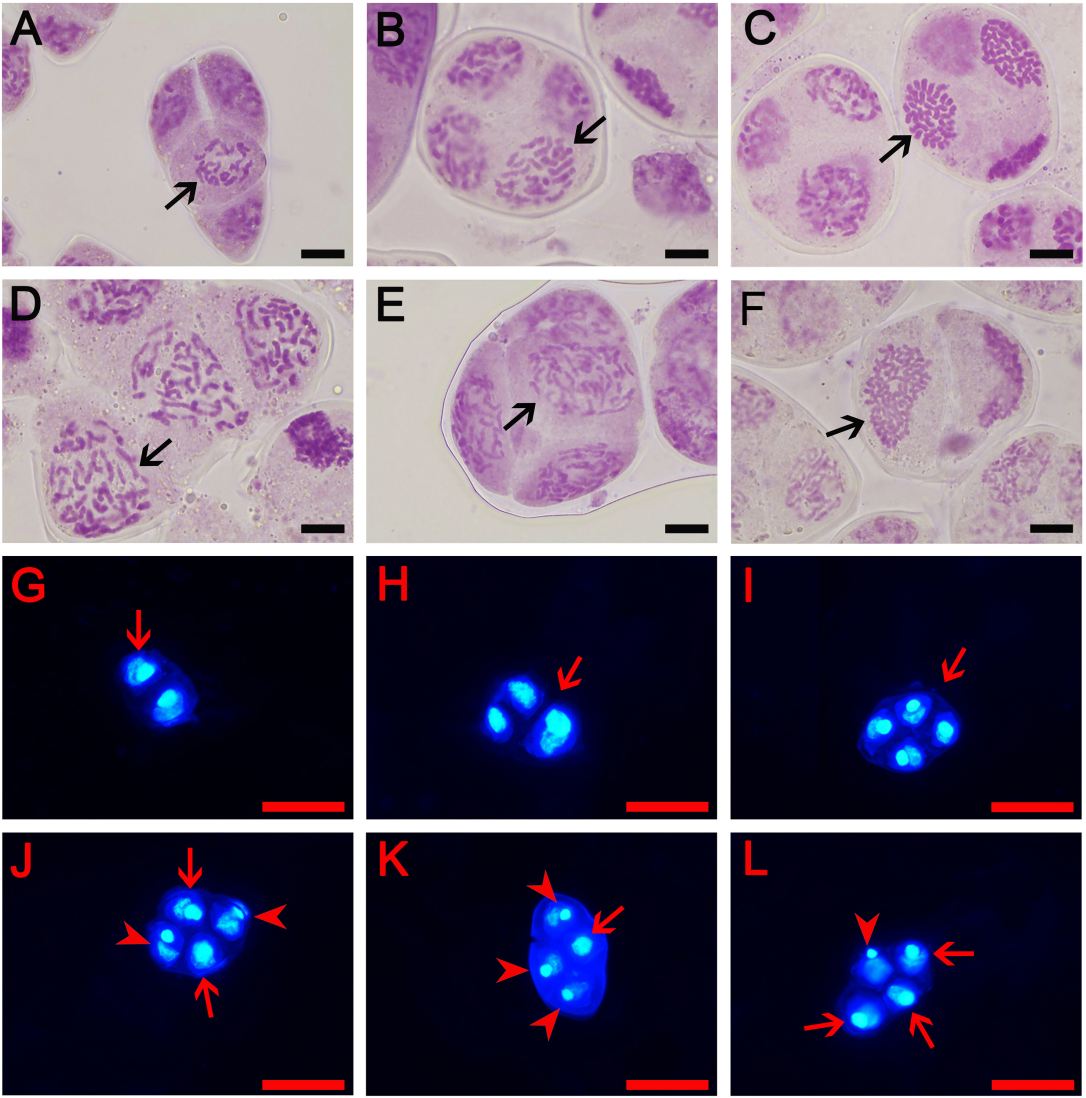
Types of male gametes in diploid and triploid cymbidiums. **(A–F)** represent early microspore stage where **(A)** The aneuploidy gamete of ‘Xiaofeng’: *x*-2 = 18 (arrow) and **(B–F)** The gamete of ‘Yuchan’: **(B)** 1*x* gamete: *x* = 20 (arrow), **(C)** Aneuploidy gamete: *x*+10 = 30 (arrow), **(D)** 2*x* gamete: 2*x* = 40 (arrow), **(E)** Aneuploidy gamete: 2*x* +2 = 42 (arrow), and **(F)** Unreduced gamete: 3*x* = 60 (arrow). Bar = 10 μm. Additionally, **(G–L)** represent mature pollens stained with 4, 6-diamidino-2-phenylindole (DAPI): **(G)** Dyad (arrow), **(H)** Triad (arrow), **(I)** Tetrad with the same size of nuclei (arrow), **(J)** Tetrad with two large (arrow) and two small nuclei (arrowhead), **(K)** Tetrad with one large (arrow) and three small nuclei (arrowhead), and **(L)** Tetrad with three large (arrow) and one small nuclei (arrowhead). Bar = 50 µm.

In order to further understand how tetraploids were formed through ‘triploid bridge’, the types of male gametes and their fertilities were examined in sexual triploid and diploid parents. Results showed that ‘Yuchan’ and ‘Huanghe’ produced 1*x*, 2*x*, 3*x* (unreduced gamete) and aneuploid male gametes (**Figures 2B–L**; **Supplementary Figure S3**). The proportion of 1*x*~2*x* aneuploid gametophytes was 77.78% and 80.00% in ‘Yuchan’ and ‘Huanghe’, respectively (**Table 3**), which proved that the main type of male gamete produced by triploids was aneuploidy. The occurrence of 1*x* and 2*x* male gametes with a rather high proportion in sexual triploids suggested that the unreduced gamete was inclined to enter into the same daughter cell during meiosis, thus resulting in the formation of 2*x* male gamete (**Figure 2J**).

**Table 3:** Type and proportion (%) of male gametes in diploid and sexual triploid cymbidiums.

Parents	The proportion of gamete (%)					
	<1*x*	1*x*	1*x*~2*x*	2*x*	2*x*~3*x*	3*x*
‘Yuchan’	0.00 ± 0.00^b^	8.89 ± 3.85^b^	77.78 ± 5.09^a^	6.67 ± 3.33^a^	3.33 ± 3.33^a^	3.33 ± 3.33^a^
‘Huanghe’	0.00 ± 0.00^b^	3.33 ± 0.00^b^	80.00 ± 10.00^a^	8.89 ± 1.92^a^	5.56 ± 3.85^a^	2.22 ± 1.92^a^
‘Xiaofeng’	18.89 ± 3.85^a^	72.22 ± 1.92^a^	7.78 ± 3.85^b^	1.11 ± 1.92^b^	0.00 ± 0.00^b^	0.00 ± 0.00^a^

Values represent mean ± standard error. Different letters behind the values within the same column indicate significant difference among cultivars based on Duncan’s multiple range test at *P* < 0.05 levels.

Diploid ‘Xiaofeng’ produced aneuploid gametes with chromosome number less than 20 (<1*x*), reduced male gametes (1*x*), aneuploid with chromosome number between 20 and 40 (1x–2x), and unreduced male gametes (2*x*) at 18.89%, 72.22%, 7.78%, and 1.11%, respectively (**Table 3**), which suggested that the main type of male gamete produced by ‘Xiaofeng’ was the reduced male gamete (1*x*).

**Figure 3:**
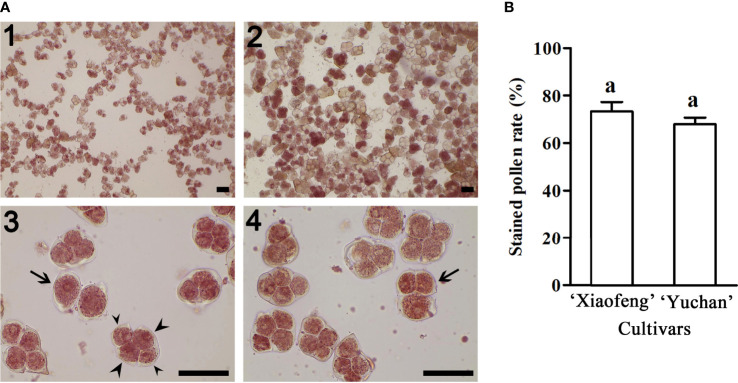
Pollen fertility of ‘Xiaofeng’ and ‘Yuchan’. **(A)** Pollen stained with 2, 3, 5-triphenyltetrazolium chloride: Pollen grains of (1) ‘Xiaofeng’ and (2) ‘Yuchan’, (3) ‘Yuchan’ pollens showed dyad (arrow) and tetrad with two small nuclei (small arrowhead) and two big nuclei (big arrowhead), and (4) ‘Yuchan’ pollens with triad (arrow). Bar=50 μm. **(B)** The percentage of stained pollens of ‘Xiaofeng’ and ‘Yuchan’, the same letter above the bars indicates no significant difference between cultivars analyzed by Duncan’s multiple range test at *P*< 0.05 level.

The viability of pollen was investigated using 2, 3, 5-triphenyltetrazolium chloride staining method. Results showed that 67.88% pollen grains of ‘Yuchan’ were stained in red, indicating their viability (**Figures 3A2, 3, 4**). Similarly, 73.32% pollen grains of ‘Xiaofeng’ were viable (**Figure 3A1**). Besides, some pollen grains with different ploidy levels were also stained in red, suggesting that all types of male gametes were fertile or partial fertile (**Figures 3A3, 4**). Moreover, there was no significant difference in the percentage of stainable pollens between the diploid and sexual triploid (**Figure 3B**).

### Meiotic abnormalities during microsporogenesis

**Figure 4:**
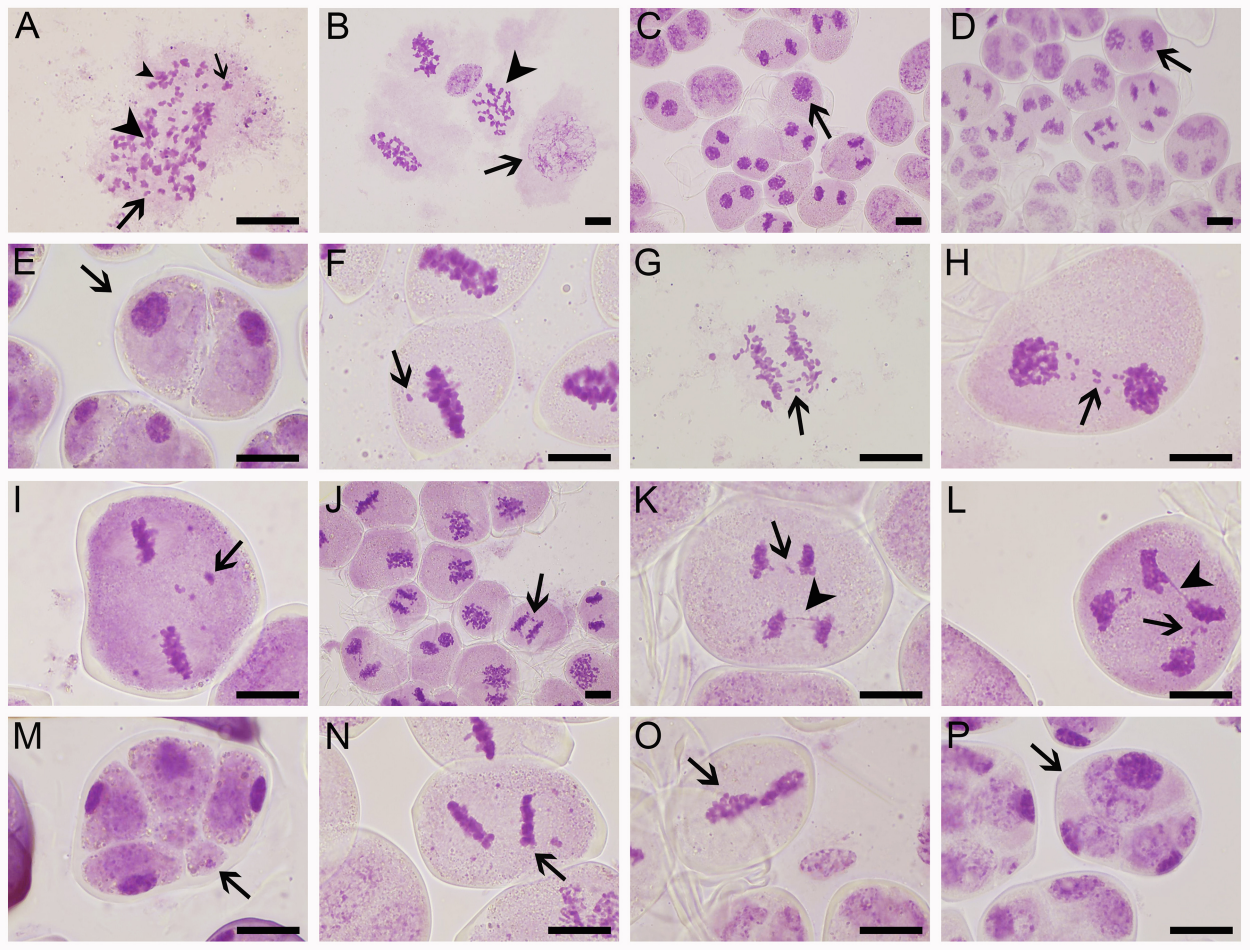
Meiotic abnormalities of sexual triploid ‘Yuchan’. **(A)** Univalent (big arrow), bivalent (small arrow), trivalent (big arrowhead), and multivalent (small arrowhead) observed at diakinesis. **(B)** Metaphase I, microspore mother cells at pachyten (arrow) or diakinesis (arrowhead) stage were observed. **(C)** Telophase I: microsporocyte failed to carry out meiosis I (arrow). **(D)** Telophase II: microsporocyte that missed meiosis I but proceeded with normal meiosis II (arrow), which resulted in the formation of dyad (arrow) **(E)**. **(F–I)** Lagging chromosomes (arrow) at metaphase I, anaphase I, telophase I, and metaphase II. **(J)** Chromosome bridge (arrow) at anaphase **(I)**. **(K)** Lagging chromosomes (arrow) and a chromosome bridge (arrowhead) at anaphase II. **(L)** Lagging chromosomes (arrow) and a chromosome bridge (arrowhead) at telophase II. **(M)** Tetrad stage: indicating micronucleus (arrow). **(N)** Metaphase II: tripolar spindles (arrow). **(O)** Metaphase II: fused spindles (arrow). **(P)** Tetrad stage, indicating triad (arrow). Bar = 20 μm.

Microsporogenesis of ‘Yuchan’, ‘Huanghe’, and ‘Xiaofeng’ were observed in order to gain a better understanding of cytological mechanisms behind the formation of different types of male gametes (**Figure 4**; **Table 4** and **Supplementary Figures S4**, **S5**). The results indicated that meiotic abnormalities included meiosis asynchrony, lagging chromosomes, chromosome bridges, and abnormal orientation of spindles during the microsporogenesis. Univalents, bivalents, trivalents, and multivalents were observed at diakinesis of ‘Yuchan’ (**Figure 4A**). At metaphase I, there were 33.0% and 24.5% microspore mother cells of ‘Yuchan’ and ‘Xiaofeng’, respectively at either diakinesis or pachytene stage (**Figure 4B**; **Table 4**). Those microspore mother cells probably missed the meiosis I (**Figure 4C**) but proceeded with normal meiosis II (**Figure 4D**), which resulted in the formation of dyads (**Figure 4E**). Lagging chromosomes were noticed at every stage of meiosis from metaphase I to telophase II (**Figures 4H, I, K, L**). Chromosome bridges were found during anaphase I and telophase I, II (**Figures 4J–L**), which might lead to the formation of aneuploid male gametes and micronuclei (**Figure 4M**). The abnormal orientation of spindles was observed during metaphase II, including tripolar spindle (**Figure 4N**) and fusion spindle (**Figure 4O**), which resulted in the formation of triads (**Figure 4P**) and dyads (**Figure 4E**). The percentage of lagging chromosomes and chromosome bridges in sexual triploids was significantly higher than that in diploid. As a result, a higher percentage of aneuploid male gametes occurred in sexual triploids (**Table 4**).

**Table 4:** Meiotic abnormalities in cymbidium ‘Yuchan’, ‘Huanghe’, and ‘Xiaofeng’.

Stage	Percentage of abnormal behavior during meiosis (%)	Parents		
		‘Yuchan’	‘Huanghe’	‘Xiaofeng’
Metaphase I	Meiotic asynchrony	33.00 ± 5.00^a^	–	24.5 ± 6.36^a^
	Lagging chromosomes	5.00 ± 1.00^a^	–	1.33 ± 0.57^b^
Anaphase I	Lagging chromosomes	21.50 ± 3.54^a^	16.00 ± 1.41^a^	5.50 ± 0.71^b^
	Chromosome bridges	4.50 ± 0.71^a^	2.00 ± 1.41^ab^	0.5 ± 0.71^b^
Telophase I	Lagging chromosomes	25.67 ± 2.89^a^	12.50 ± 3.54^b^	2.5 ± 0.71^c^
	Meiotic asynchrony	26.00 ± 6.08^ab^	32.33 ± 0.58^a^	18.00 ± 1.41^b^
Metaphase II	Lagging chromosomes	8.33 ± 1.15^a^	7.50 ± 0.71^a^	4.00 ± 1.41^b^
	Tripolar spindles	7.00 ± 1.00^a^	5.50 ± 0.71^ab^	3.00 ± 1.41^b^
	Fused spindles	1.67 ± 0.58^a^	0.67 ± 0.58^ab^	0.00 ± 0.00^b^
Anaphase II	Lagging chromosomes	26.67 ± 3.06^a^	19.33 ± 1.53^b^	16.33 ± 2.52^b^
	Chromosome bridges	9.33 ± 1.53^a^	5.33 ± 0.57^b^	0.00 ± 0.00^b^
Telophase II	Lagging chromosomes	16.67 ± 1.53^a^	15.33 ± 2.31^ab^	12.33 ± 0.47^b^
	Chromosome bridges	6.33 ± 1.53^a^	1.33 ± 0.58^b^	2.67 ± 0.94^b^
	Meiotic asynchrony	30.67 ± 0.58^a^	21.67 ± 2.08^b^	12.00 ± 2.83^c^
Tetrad period	Micronuclei	8.30 ± 1.53^a^	8.67 ± 1.53^a^	3.00 ± 1.00^b^
	Dyad	3.93 ± 0.06^a^	2.42 ± 0.09^b^	1.40 ± 0.33^b^
	Triad	3.76 ± 0.04^a^	3.33 ± 0.54^a^	0.58 ± 0.19^b^

“-” indicates that data were not collected. Values represent mean ± standard error. Different letters within the same row indicate significant differences among cultivars based on Duncan’s multiple range test at P< 0.05 level.

### Statistical analysis of survival rate of plantlets with different ploidy levels

**Figure 5:**
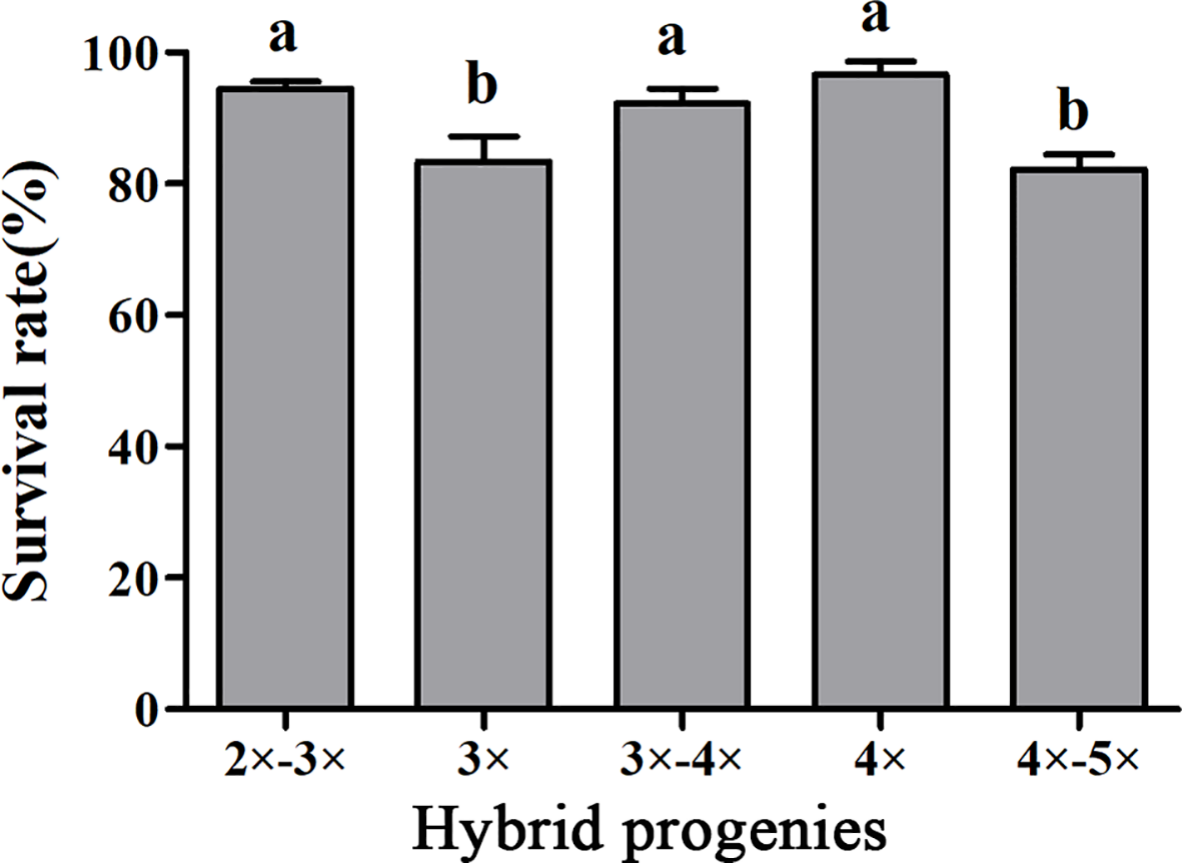
Survival rates of hybrid seedlings with different ploidy levels after being transplanted to plastic bags containing a substrate and grown in a shaded greenhouse. Bars represent standard error, and different letters on the top of bars indicate significant difference in survival rates among hybrid progenies analyzed by Duncan’s multiple range test at *P*< 0.05 level.

To determine the survivability of plantlets, the survival rates of plantlets with different ploidy levels grown in the shaded greenhouse were evaluated. The survival rate of tetraploid plants was 96.67%, which was significantly higher than that of triploids. Interestingly, the survival rates of 2×~3× and 3×~4× aneuploid plants were also significantly higher than that of triploids. There was no significant difference in the survival rate between 4×~5× aneuploid and triploid plants (**Figure 5**).

## Discussion

Polyploidization is considered as an important evolutionary force. The most common mechanism of polyploid origin is believed to be through production of unreduced gametes (Clo et al., 2022). There are two main models explaining the pathways of polyploid formation in diploid populations: (i) frequency-dependent minority cytotype exclusion (Levin, 1975; Husband, 2000) and (ii) the ‘triploid bridge’ hypothesis (Ramsey and Schemske, 1998; Burton and Husband, 2001; Husband, 2004; Peckert and Chrtek, 2006; Schinkel et al., 2017). According to the ‘triploid bridge’ hypothesis, triploids are first formed *via* the union of reduced and unreduced gametes. Subsequently, backcrosses of triploids to diploids or crosses between triploids can generate tetraploids (Bretagnolle and Thompson, 1995; Ramsey and Schemske, 1998). In the present study, we documented that sexual triploid cymbidiums produced functional 1*x*, 2*x*, 3*x*, and aneuploid gametes after backcross to diploids or through the cross between triploids. We further showed the triploid cross resulted in the formation of tetraploids at a high percentage and also pentaploids. Our results demonstrate that the sexual triploids act as a bridge for efficiently producing tetraploid and even polyploid with higher ploidy level in *Cymbidium*.

The production of functional *x*, 2*x*, 3*x*, and aneuploid gametes is important for triploids to fulfil the role as a bridge (Burton and Husband, 2001; Peckert and Chrtek, 2006; Marasek-Ciolakowska et al., 2014; Li et al., 2017; Zhang et al., 2017; Trankner et al., 2020). The percentages in occurrence of different types of gametes depended on plant species, origin of triploids, and gamete types (Marasek-Ciolakowska et al., 2014; Geng et al., 2019; Trankner et al., 2020). In general, there was a low frequency in occurrence of *x*, 2*x*, and 3*x* gametes but a high frequency with aneuploid gametes in triploids. For example, the percentages of *x*, 2*x*, and aneuploid gametes produced by triploid *Datura stramonium* were 2.6%, 1.2%, and 96.2%, respectively (Satina and Blakeslee, 1937) and by triploid *Pennisetum glaucum* were 1.85%, 1.85%, and 96.3%, respectively (Dujardin and Hanna, 1988). The percentages of *x* and 2*x* in autotriploid cucumber were 1.44% and 1.44% (Diao et al., 2009). Our results primarily concurred with the above reports and showed that the percentages of *x*, 2*x*, 3*x*, and aneuploid male gametes in ‘Yuchan’ were 8.89%, 6.67%, 3.33%, and 81.11%, respectively and in ‘Huanghe’ were 3.33%, 8.89%, 2.22%, and 85.56% (**Table 3**). The occurrence of 2*x* gametes in triploids is critical as it allows to the establishment of balanced tetraploid progenies from 3*x*-4*x* (Vuylsteke et al., 1993; Ramsey and Schemske, 1998; Ramanna and Jacobsen, 2003) or 3*x*-3*x* crosses demonstrated in this study.

Duo to the formation of functional gametes, triploids may produce tetraploid offspring through backcrosses with diploids or crossing with other triploids (Husband, 2004). In *Hieracium echioides*, the cross of 2× × 3× resulted in largely diploid progenies (92%); while in the cross of 3× × 2×, 56% hybrids were tetraploids, and the cross of 3× × 3× produced 60% tetraploids, 26% pentaploids, and 7% hexaploids (Peckert and Chrtek, 2006). In *Tulip*, one tetraploid and four pentaploids were produced in 3× × 2× crosses. In contrast, no tetraploids were obtained in 2× × 3× and 3× × 3× crosses (Marasek-Ciolakowska et al., 2014). In *Populus*, however, a cross of 2× × 3× produced 4% tetraploid hybrids (Wang et al., 2017). In *Echinacea purpurea*, tetraploids were generated in both 2× × 3× and 3× × 2× crosses (Li et al., 2017), while in *Phegopteris decursivepinnata*, both tetraploid and pentaploid were formed in the 3× × 2× cross (Nakato and Masuyama, 2021). These results showed that tetraploids were produced more frequently in crosses of 3× × 2× and 3× × 3× than that of 2× × 3×. Our results indicated that albeit tetraploids were formed in combinations of diploid × triploid, however, the frequency (2.08%) was low. On the contrary, the percentage of tetraploids in the hybrids of triploid × triploid was very high (52.5%), suggesting that hybridization between sexual triploids could be a principal way of producing tetraploid through ‘triploid bridge’ in *Cymbidium*.

In theory, triploids are sterility due to the unbalanced meiotic chromosome segregation, which resulted in the production of aneuploid gametes (Kohler et al., 2010; Wang et al., 2017; Zhang et al., 2017). But in practice, a lot of triploids can produce functional euploid gametes, especially for *x* and 2*x* gametes in different proportions, which can be used as male or female parent in cross breeding programs (Lim et al., 2003; Hayashi et al., 2009; Zhou et al., 2009; Nakato and Masuyama, 2021). Why does a triploid produce euploid gametes and why is it regarded as a bridge in polyploid evolution? Thus far, little information is available to the questions. Here we propose a hypothesis of coordinate actions of unreduced gamete to address the questions: During meiosis of a sexual polyploid, two chromosome sets of the 2*n* gamete are inclined to be assorted to a daughter cell, resulting in the production of 2*x* gamete, such a chromosome behavior during meiosis mainly depends on the genetic relationship of the parents who provide the chromosome set. When the genetic relationship is very close, such as sexual autopolyploid, the main chromosome pairing configuration at diakinesis is trivalent (sexual autotriploid) or quadrivalent (sexual autotetraploid); when the genetic relationship is far different, such as sexual allopolyploid, the main chromosome pairing configuration is a univalent and a bivalent (sexual allotriploid) or two bivalents (sexual allotetraploid). In fact, the meiotic configuration 8I+8II+2III was the most common in two natural triploid populations of *Campuloclinium macrocephalum*, and their pollen fertilities were 44.74 and 52.69%, respectively (Farco and Dematteis, 2014). Similar results were obtained in allotriploid *P. alba* × *P. berolinensis* ‘Yinzhong’ (Wang et al., 2017). Lavia et al. (2011) reported that the main chromosome paring configuration in sexual autotriploid *Arachis pintoi* was trivalent, and the pollen grain viability was 42.47%. Ramanna et al. (2003) reported that most *Alstroemeria* interspecific F_1_ hybrids of Chilean-Brazilian species simultaneously produced 2*n* male and female gametes; and all the F_2_ progeny plants, which were resulted from self-pollination of the F_1_ hybrids, were typical allotetraploids. Additionally, most of them formed 16 bivalents and a small proportion formed multivalents during metaphase I stages of meiosis. Triploids that originated through the fusion of 2*n* × *n* gametes of the same taxon showed more regular meiotic behavior and higher fertility than triploids from the contact zone of diploids and tetraploids or triploids of hybrid origin (Kovalsky et al., 2018). Natural *Dactylis* polyploids exhibited successful chromosome pairing during meiosis, whereas artificial polyploids did not, suggesting that there was a selection for sexual fertility in order to stabilize meiosis in natural polyploids (Lumaret and Retired, 1988). Our results indicated that both sexual triploids ‘Yuchan’ and ‘Huanghe’ produced *x* and 2*x* male gamete (**Figure 2J**) with 2*x* gamete frequencies at 6.67% and 8.89%, respectively, and the percentage of viable pollen was 67.88% in ‘Yuchan’. These results further proved that two chromosome sets of the 2*n* gamete were inclined to be assorted to a daughter cell. However, due to the sophisticated origin of 2*n* gamete and sexual triploid, the chromosome paring configuration was not typical and the occurrence percentage of 2*x* and *x* gametes was not high. Nevertheless, due to the occurrence in 2*n* gametes, triploids play an important role in polyploid evolution.

**Figure 6:**
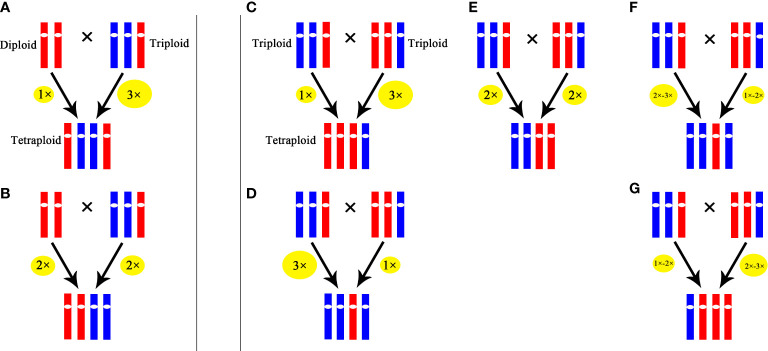
Possible pathways for producing tetraploid through ‘triploid bridge’. **(A)** and **(B)** represent possible pathways in the cross of 2××3×. **(C-G)** represent possible pathways in the cross of 3××3×. The size of ellipse represents ploidy level of gamete, the larger the size is, the higher the ploidy level is.

Our results indicated that in the cross of 2*×* × 3*×*, 25% hybrids were triploids, which was similar to the results in *Tulip* (Marasek-Ciolakowska et al., 2014) and *Populus* (Wang et al., 2017). These triploids were probably formed by the fusion of a 2*x* male gamete produced by the triploid with a haploid female gamete from the diploid or originated from the fusion of a haploid male gamete from the triploid with unreduced female gamete from the diploid. A tetraploid was also obtained in the cross of 2× × 3×, which was likely formed by the fusion of an unreduced male gamete (3*x*) produced by the triploid with a haploid female gamete from the diploid or originated from the fusion of 2*x* male gamete from the triploid with unreduced female gamete from the diploid. In the cross of 3× × 3×, 52.5% progenies were tetraploids, and these tetraploids probably originated from the fusion of an unreduced gamete produced by one parent with a haploid gamete from the other or the fusion of two 2*x* gamete from the parents. Similar results were reported in *H. echioides* where 3× × 3× produced 60% tetraploid (Peckert and Chrtek, 2006). Because of the low percentage of occurrence in euploid gametes in triploid, the aneuploid gametes might play an important role in the production of tetraploids. The possible pathways of producing tetraploids through ‘triploid bridge’ in *Cymbidium* are illustrated in **Figure 6**.

A long-standing problem in polyploid breeding is triploid block, which is a reproductive barrier caused by malfunction of endosperm. The endosperm supports the development of the embryo by providing nutrients for its growth, and the genetic relationship between the endosperm and the embryo is important for higher plants evolution (Johnston et al., 1980; Koehler et al., 2010; Stoute et al., 2012; Huc et al., 2022). In the majority of plants, the maternal and paternal chromosome dosages in the endosperm are considered to be critical for seed development and fertility (Vinkenoog et al., 2003; Koehler et al., 2010; Schinkel et al., 2017). However, orchid seeds have rudimentary embryo and lack of endosperm (Yeung, 2017; Chen et al., 2018). Seed germination and seedling establishment depend on the successful interaction between protocorms and mycorrhizal fungi either *in vitro* or *ex vitro*, thus endosperm is not a limiting factor affecting seed germination (Chugh et al., 2009; Xu et al., 2011; Yeung, 2017; Yeh et al., 2019; Bhatti and Thakur, 2022). Our study showed that hybrid seeds derived from the crosses of 3× × 2×, 2× × 3×, and 3× × 3× were well developed and able to germinate normally on culture medium. Plantlets with different ploidy levels grew healthy in greenhouse. Thus, triploid block is not a concern in *Cymbidium* as sexual triploids can produce seeds through hybridization with appropriate parents and the seeds can readily germinate.

*Cymbidium* is a renowned genus in the orchid family that distributed in tropical and subtropical areas of Asia, Papua New Guinea, and Australia (Ai et al., 2021). It exhibits distinctive ecological diversification and occurs in terrestrial, epiphytic, lithophytic, and saprophytic life forms (Yukawa and Stern, 2002; Ning et al., 2018). The cross compatibility between different species was reported to be high, and even an increasing number of interspecific and intergeneric hybrids has been obtained through artificial pollination (Li et al., 2014; Ogura-Tsujita et al., 2014; Joffard et al., 2022). These interspecific hybrids have a high percentage in occurrence of 2*n* gametes and have been successfully employed to create sexual polyploids in breeding programs (Zeng et al., 2020; Kondo et al., 2022). However, because of reproductive isolation caused by geographical, temporal, and spatial isolations, the interspecific hybrids are rare in nature. The triploid crossability and 2*n* gamete formation demonstrated in the present study may represent a viable way for creating new polyploid cymbidium through hybridization.

## Conclusion

As far as is known, this is the first investigation of the crossability of sexual triploids in cymbidiums. Our study documented that both triploid ‘Yuchan’ and ‘Huanghe’ were fertile and able to be used as male or female parents in cross breeding. Sexual triploid cymbidiums produced fertile male gametes of 1*x*, 1*x*~2*x*, 2*x*, 2*x*~3*x*, and 3*x*. The production of 2*x* male gametes could be resulted from the probability that two chromosome sets of the 2*n* gamete were inclined to enter into the same daughter cell during meiosis. The cross of diploid × triploid generated diploid, triploid, and tetraploid with frequencies at 2.1%, 25.0%, and 2.1%, respectively. The cross of triploid × triploid produced tetraploid and pentaploid hybrids with proportions of 52.5% and 2.5%, respectively. The survival rate of tetraploid was significantly higher than that of triploid. Our results indicate that the triploid cymbidiums are not reproductive barrier but act as a bridge in the formation of polyploid plants. A hypothesis of coordinate actions of unreduced gamete was proposed to explain why sexual triploids produce 1*x* and 2*x* euploid gametes. Further research with more sexual polyploids including auto and allopolyploids, along with the use of genomic *in situ* hybridization (GISH) should be conducted to test this hypothesis.

## Data availability statement

The original contributions presented in the study are included in the article/**Supplementary Material**. Further inquiries can be directed to the corresponding authors.

## Author contributions

Z-SZ and R-ZZ designed the study. M-ML, R-ZZ, LX, H-RG, and J-RZ performed the experiment. M-ML, R-ZZ, and LX carried out and analyzed the data. M-ML and Q-LS wrote a first draft of the manuscript that was further critically reviewed and edited by JC, R-ZZ, LX, QW, H-RG, and J-RZ. All authors contributed to the article and approved the submitted version.

## Funding

This work was supported by the Research and Development Plan in Key Area of Guangdong Province, China (2022B0202080004), Special Project of Agriculture Science Independent Innovation of Guangzhou Agricultural Bureau (21102422), Science and Technology Program of Guangzhou (202002030068) and Project of Guangdong Provincial Key Laboratory of Plant Molecular Breeding (GPKLPMB202206).

## Conflict of interest

The authors declare that the research was conducted in the absence of any commercial or financial relationships that could be construed as a potential conflict of interest.

## Publisher’s note

All claims expressed in this article are solely those of the authors and do not necessarily represent those of their affiliated organizations, or those of the publisher, the editors and the reviewers. Any product that may be evaluated in this article, or claim that may be made by its manufacturer, is not guaranteed or endorsed by the publisher.

## Supplementary material

Supplementary Figure 1 Cymbidium cultivars used for hybridization.

Supplementary Figure 2 Morphology of seeds, test-tube seedlings and transplanted seedlings.

Supplementary Figure 3 Types of male gametes at early microspore stage in diploid and triploid Cymbidium.

Supplementary Figure 4 Meiotic abnormalities of sexual triploid ‘Huanghe’.

Supplementary Figure 5 Meiotic abnormalities of diploid ‘Xiaofeng’.

## References

[B1] Ai Y. Li Z. Sun W. H. Chen J. Zhang D. Y. Ma L.. (2021). The *Cymbidium* genome reveals the evolution of unique morphological traits. Hortic. Res. 8, 255. doi: 10.1038/s41438-021-00683-z 34848682 PMC8633000

[B2] Alexander L. (2020). Ploidy level influences pollen tube growth and seed viability in interploidy crosses of *Hydrangea macrophylla. Front* . Plant Sci. 11. doi: 10.3389/fpls.2020.00100 PMC7042312 32140167

[B3] Bergstrom I. (1940). On the progeny of diploid × triploid *Populus tremula* with special reference to the occurrence of tetraploidy. Hereditas 26, 191–201. doi: 10.1111/j.1601-5223.1940.tb03231.x

[B4] Bhatti S. K. Thakur M. (2022). An overview on orchids and their interaction with endophytes. Bot. Rev. doi: 10.1007/s12229-022-09275-5

[B5] Bretagnolle F. Thompson J. D. (1995). Gametes with the somatic chromosome number: mechanisms of their formation and role in the evolution of autopolyploid plants. New Phytol. 129, 1–22. doi: 10.1111/j.1469-8137.1995.tb03005.x 33874422

[B6] Burton T. L. Husband B. C. (2001). Fecundity and offspring ploidy in matings among diploid, triploid and tetraploid *Chamerion angustifolium* (Onagraceae): consequences for tetraploid establishment. Heredity 87, 573–582. doi: 10.1046/j.1365-2540.2001.00955.x 11869348

[B7] Cai J. Liu X. Vanneste K. Proost S. Tsai W. Liu K. W.. (2015). The genome sequence of the orchid *Phalaenopsis equestris* . Nat. Genet. 47, 65–72. doi: 10.1038/ng.3149 25420146

[B8] Chen Y. Zhang C. Wang X. F. Ao C. Q. (2018). Fertilisation of polar nuclei and formation of early endosperms in *Dendrobium catenatum*: evidence for the second fertilisation in orchidaceae. Aust. J. Bot. 66, 354–359. doi: 10.1071/BT17211

[B9] Chugh S. Guha S. Rao I. U. (2009). Micropropagation of orchids: a review on the potential of different explants. Sci. Hortic. 122, 507–520. doi: 10.1016/j.scienta.2009.07.016

[B10] Clo J. Padilla-Garcia N. Kolar F. (2022). Polyploidization as an opportunistic mutation: The role of unreduced gametes formation and genetic drift in polploid establishment.J. Evol. Biol. 35, 1099–1109. doi: 10.1111/jeb.14055 35770884

[B11] Cui J. Chen J. J. Henny R. (2009). Regeneration of aeschynanthus radicans *via* direct somatic embryogenesis and analysis of regenerants with flow cytometry. *In vitro cell* . Dev. Biol.-Plant. 45, 34–43. doi: 10.1007/s11627-008-9147-9

[B12] Czarnecki D. M. Hershberger A. J. Robacker C. D. Clark D. G. Deng Z. A. (2014). Ploidy levels and pollen stainability of *Lantana camara* cultivars and breeding lines. HortScience 49, 1271–1276. doi: 10.21273/HORTSCI.49.10.1271

[B13] Diao W. P. Bao S. Y. Jiang B. Cui L. Chen J. F. (2009). Primary trisomics obtained from autotriploid by diploid reciprocal crosses in cucumber. Sex Plant Reprod. 22, 45–51. doi: 10.1007/s00497-008-0090-z 20033455

[B14] Dujardin M. Hanna W. W. (1988). Cytology and breeding behavior of a partially fertile triploid pearl millet. J. Hered. 79, 216-218. doi: 10.1093/oxfordjournals.jhered.a110499

[B15] Duszynska D. Vilhjalmsson B. Bravo R. C. Swamidatta S. Juenger T. E. Donoghue M. T. A.. (2019). Transgenerational effects of inter-ploidy cross direction on reproduction and F2 seed development of *Arabidopsis thaliana* F1 hybrid triploids. Plant Reprod. 32, 275–289. doi: 10.1007/s00497-019-00369-6 30903284 PMC6675909

[B16] Ehlenfeldt M. K. Ortiz R. (1995). Evidence on the nature and origins of endosperm dosage requirements in *Solanum* and other angiosperm genera. Sex Plant Reprod. 8, 189–196. doi: 10.1007/BF00228936

[B17] Erilova A. Brownfield L. Exner V. Rosa M. Twell D. Scheid O. M.. (2009). Imprinting of the polycomb group gene *MEDEA* serves as a ploidy sensor in *Arabidopsis* . PLoS Genet. 5, e1000663. doi: 10.1371/journal.pgen.1000663 19779546 PMC2738949

[B18] Farco G. E. Dematteis M. (2014). Meiotic behavior and pollen fertility in triploid and tetraploid natural populations of *Campuloclinium macrocephalum* (Eupatorieae, asteraceae). Plant Syst. Evol. 30, 1843–1852. doi: 10.1007/s00606-014-1011-2

[B19] Friedman W. E. Madrid E. N. Williams J. H. (2008). Origin of the fittest and survival of the fittest: Relating female gametophyte development to endosperm genetics. Int. J. Plant Sci. 169, 79–92. doi: 10.1086/523354

[B20] Geng X. N. Han Z. Q. Yang J. Du K. Han Q. Kang X. Y. (2019). The different origins of artificially-induced unreduced female gametes and their effect on transmitted parental heterozygosity in *Populus* . Euphytica 215, 181. doi: 10.1007/s10681-019-2501-7

[B21] Haig D. (2013). Kin conflict in seed development: an interdependent but fractious collective. Annu. Rev. Cell Dev. Biol. 29, 189–211. doi: 10.1146/annurev-cellbio-101512-122324 23641801

[B22] Hayashi M. Kato J. Ohashi H. Mii M. (2009). Unreduced 3× gamete formation of allotriploid hybrid derived from the cross of *Primula denticulata* (4×) x *P. rosea* (2×) as a causal factor for producing pentaploid hybrids in the backcross with pollen of tetraploid *P. denticulata* . Euphytica 169, 123–131. doi: 10.1007/s10681-009-9955-y

[B23] Hedren M. Lorenz R. Teppner H. Dolinar B. Giotta C. Griebl N.. (2018). Evolution and systematics of polyploid *Nigritella* (Orchidaceae). Nord. J. Bot. 36, e01539. doi: 10.1111/njb.01539

[B24] Henry I. M. Dilkes B. P. Young K. Watson B. Wu H. Comai L. (2005). Aneuploidy and genetic variation in the *Arabidopsis thaliana* triploid response. Genetics 170, 1979–1988. doi: 10.1534/genetics.104.037788 15944363 PMC1449780

[B25] Huang W. T. Fang Z. M. Zeng S. J. Zhang J. X. Wu K. L. Chen Z. L.. (2012). Molecular cloning and functional analysis of three *FLOWERING LOCUS T (FT)* homologous genes from Chinese *Cymbidium* . Int. J. Mol. Sci. 13, 11385–11398. doi: 10.3390/ijms130911385 23109860 PMC3472752

[B26] Huc J. Dziasek K. Pachamuthu K. Woh T. Kohler C. Borges F. (2022). Bypassing reproductive barriers in hybrid seeds using chemically induced epimutagenesis. Plant Cell 34, 989–1001. doi: 10.1093/plcell/koab284 34792584 PMC8894923

[B28] Husband B. C. (2000). Constraints on polyploid evolution: a test of the minority cytotype exclusion principle. Proc. R. Lond. 267, 217–223. doi: 10.1098/rspb.2000.0990 PMC1690524 10714875

[B27] Husband B. C. (2004). The role of triploid hybrids in the evolutionary dynamics of mixed-ploidy populations. Biol. J. Linn. Soc 82, 537–546. doi: 10.1111/j.1095-8312.2004.00339.x

[B29] Jike W. Li M. G. Zadra N. Barbaro E. Sablok G. Bertorelle G.. (2020). Phylogenomic proof of recurrent demipolyploidization and evolutionary stalling of the “Triploid bridge” in arundo (Poaceae). Int. J. Mol. Sci. 21, 5247. doi: 10.3390/ijms21155247 32722033 PMC7432733

[B30] Joffard N. Olofsson C. Friberg M. Sletvold N. (2022). Extensive pollinator sharing does not promote character displacement in two orchid congeners. Evolution 76, 749–764. doi: 10.1111/evo.14446 35188979

[B31] Johnston S. A. den Nijs T. P. Peloquin S. J. Hanneman R. E. J. (1980). The significance of genic balance to endosperm development in intraspecific crosses. Theor. Appl. Genet. 57, 5–9. doi: 10.1007/BF00276002 24302359

[B32] Jones K. D. Reed S. M. (2007). Analysis of ploidy level and its effects on guard cell length, pollen diameter, and fertility in *Hydrangea macrophylla* . HortScience 42, 483–488. doi: 10.21273/HORTSCI.42.3.483

[B33] Koehler C. Scheid O. M. Erilova A. (2010). The impact of the triploid block on the origin and evolution of polyploid plants. Trend. Genet. 26, 142–148. doi: 10.1016/j.tig.2009.12.006 20089326

[B34] Kondo H. Deguchi A. Kikuchi S. Miyoshi K. (2022). Two pathways of 2*n* gamete formation and differences in the frequencies of 2*n* gametes between wild species and interspecific hybrids. Plant Cell Rep. doi: 10.1007/s00299-022-02915-5 35984498

[B35] Kovalsky I. E. Luque J. M. R. Elias G. Fernandez S. A. Neffa V. G. S. (2018). The role of triploids in the origin and evolution of polyploids of *Turnera sidoides* complex (Passifloraceae, turneroideae). J. Plant Res. 131, 77–89. doi: 10.1007/s10265-017-0974-9 28831641

[B36] Lavia G. I. Ortiz A. M. Robledo G. Fernandez A. Seijo G. (2011). Origin of triploid *Arachis pintoi* (Leguminosae) by autopolyploidy evidenced by FISH and meiotic behaviour. Ann. Bot. 108, 103–111. doi: 10.1093/aob/mcr108 21693666 PMC3119619

[B37] Levin D. A. (1975). Minority cytotype exclusion in local plant populations. Taxon 24, 35–43. doi: 10.2307/1218997

[B38] Li Q. L. Jiang W. Z. Ren Y. Chen R. Li X. L. Yang Y. S.. (2017). *In vitro* cloning potential and phytochemical evaluations of aneuploid individuals produced from reciprocal crosses between diploid and triploid in *Echinacea purpurea* l. Acta Soc Bot. Pol. 86, 1-16. doi: 10.5586/asbp.3556

[B40] Lim K. B. Ramanna M. S. Jacobsen E. van Tuyl J. M. (2003). Evaluation of BC2 progenies derived from 3x-2x and 3x-4x crosses of *Lilium* hybrids: a GISH analysis. Theor. Appl. Genet. 106, 568–574. doi: 10.1007/s00122-002-1070-6 12589558

[B41] Lin B. Y. (1984). Ploidy barrier to endosperm development in maize. Genetics 107, 103–115. doi: 10.1007/BF00056440 17246209 PMC1202307

[B39] Li X. B. Xiang L. Wang Y. Luo J. Wu C. Sun C. B.. (2014). Genetic diversity, population structure, pollen morphology and cross-compatibility among Chinese cymbidiums. Plant Breed. 133, 145–152. doi: 10.1111/pbr.12125

[B42] Lumaret R. Retired M. B. (1988). Cytology, genetics and evolution in the genus *Dactylis* . Crit. Rev. Plant Sci. 7, 55–91. doi: 10.1080/07352688809382259

[B43] Marasek-Ciolakowska A. Xie S. L. Arens P. van Tuyl J. M. (2014). Ploidy manipulation and introgression breeding in Darwin hybrid tulips. Euphytica 198, 389–400. doi: 10.1007/s10681-014-1115-3

[B44] Moura Y. A. Alves-Pereira A. da Silva C. C. Souza L. M. de Souza A. P. Koehler S. (2020). Secondary origin, hybridization and sexual reproduction in a diploid-tetraploid contact zone of the facultatively apomictic orchid *Zygopetalum mackayi* . Plant Biol. 22, 939–948. doi: 10.1111/plb.13148 32558140

[B45] Nakato N. Masuyama S. (2021). Polyploid progeny from triploid hybrids of *Phegopteris decursivepinnata* (Thelypteridaceae). J. Plant Res. 134, 195–208. doi: 10.1007/s10265-021-01255-x 33559786

[B46] Ning H. J. Ao S. Y. Fan Y. R. Fu J. X. Xu C. M. (2018). Correlation analysis between the karyotypes and phenotypic traits of Chinese cymbidium cultivars. Hortic. Environ. Biote. 59, 93–103. doi: 10.1007/s13580-018-0010-6

[B47] Ogura-Tsujita Y. Miyoshi K. Tsutsumi C. Yukawa T. (2014). First flowering hybrid between autotrophic and mycoheterotrophic plant species: breakthrough in molecular biology of mycoheterotrophy. J. Plant Res. 127, 299–305. doi: 10.1007/s10265-013-0612-0 24310615

[B48] Okamoto T. Ohnishi Y. Toda E. (2017). Development of polyspermic zygote and possible contribution of polyspermy to polyploid formation in angiosperms. J. Plant Res. 130, 485–490. doi: 10.1007/s10265-017-0913-9 28275885

[B49] Peckert T. Chrtek J. (2006). Mating interactions between coexisting diploid, triploid and tetraploid cytotypes of *Hieracium echioides* (*Asteraceae*). Folia Geobot. 41, 323–334. doi: 10.1007/BF02904945

[B50] Ramanna M. S. Jacobsen E. (2003). Relevance of sexual polyploidization for crop improvement - a review. Euphytica 133, 3–18. doi: 10.1023/A:1025600824483

[B51] Ramanna M. S. Kuipers A. G. J. Jacobsen E. (2003). Occurrence of numerically unreduced (2n) gametes in *Alstroemeria* interspecific hybrids and their significance for sexual polyploidisation. Euphytica 133, 95–106. doi: 10.1023/A:1025652808553

[B52] Ramsey J. Schemske D. W. (1998). Pathways, mechanisms, and rates of polyploid formation in flowering plants. Annu. Rev. Ecol. Syst. 29, 467–501. doi: 10.1146/annurev.ecolsys.29.1.467

[B53] Satina S. Blakeslee A. F. (1937). Chromosome behavior in triploids of *Datura* stramonium. i. the male gametophyte. Am. J. Bot. 24, 518–27. doi: 10.2307/2437074

[B54] Schinkel C. C. F. Kirchheimer B. Dullinger S. Geelen D. De Storme N. Hoerandl E. (2017). Pathways to polyploidy: indications of a female triploid bridge in the alpine species *Ranunculus kuepferi* (Ranunculaceae). Plant Syst. Evol. 303, 1093–1108. doi: 10.1007/s00606-017-1435-6 29081576 PMC5640749

[B55] Scott R. J. Spielman M. Bailey J. Dickinson H. G. (1998). Parent-of-origin effects on seed development in *Arabidopsis thaliana* . Development 125, 3329–3341. doi: 10.1242/dev.125.17.3329 9693137

[B56] Stoute A. I. Varenko V. King G. J. Scott R. J. Kurup S. (2012). Parental genome imbalance in *Brassica oleracea* causes asymmetric triploid block. Plant J. 71, 503–516. doi: 10.1111/j.1365-313X.2012.05015.x 22679928

[B57] Thakur S. Dutt H. C. (2021). *Cymbidium macrorhizon* lindl. (Orchidaceae): a new record for flora of jammu and Kashmir, India. Natl. Acad. Sci. Lett. 44, 271–274. doi: 10.1007/s40009-020-00985-1

[B58] Thompson J. D. Lumaret R. (1992). The evolutionary dynamics of polyploid plants: origins, establishment and persistence. Trends Ecol. Evol. 7, 302–307. doi: 10.1016/0169-5347(92)90228-4 21236040

[B59] Trankner C. Gunther K. Sahr P. Engel F. Hohe A. (2020). Targeted generation of polyploids in *Hydrangea macrophylla* through cross-based breeding. BMC Genet. 21, 147. doi: 10.1186/s12863-020-00954-z 33287693 PMC7720383

[B60] Vilcherrez-Atoche J. A. Iiyama C. M. Cardoso J. C. (2022). Polyploidization in orchids: from cellular changes to breeding applications. Plants 11, 469. doi: 10.3390/plants11040469 35214806 PMC8874786

[B61] Vinkenoog R. Bushell C. Spielman M. Adams S. Dickinson H. G. Scott R. J. (2003). Genomic imprinting and endosperm development in flowering plants. Mol. Biotechnol. 25, 149–184. doi: 10.1385/MB:25:2:149 14526125

[B62] Vuylsteke D. Ortiz R. Pasberg-Gauhl C. Gauhl F. Speijer P. (1993). Plantain and banana research at the international research institute of tropical agriculture. HortScience 28873, 970–971. doi: 10.21273/HORTSCI.28.9.874

[B63] Wang J. Huo B. B. Liu W. T. Li D. L. Liao L. (2017). Abnormal meiosis in an intersectional allotriploid of *Populus* l. and segregation of ploidy levels in 2× x 3×progeny. PLoS One 12, e0181767. doi: 10.1371/journal.pone.0181767 28732039 PMC5521839

[B64] Wang J. Kang X. Y. Zhu Q. (2010). Variation in pollen formation and its cytological mechanism in an allotriploid white poplar. Tree Genet. Genomes 6, 281–290. doi: 10.1007/s11295-009-0248-3

[B65] Xie L. Ke L. Z. Lu X. Q. Chen J. J. Zhang Z. S. (2022). Exploiting unreduced gametes for improving ornamental plants. Front. Plant Sci. 13. doi: 10.3389/fpls.2022.883470 PMC9207335 35734261

[B66] Xu X. W. Cai M. L. Yang Y. P. Peng K. K. Zeng A. P. Jiang N.. (2011). Hybridization and *in vitro* seed germination of *Cymbidium kanran* (in Chinese). Acta Hortic. Sin. 38, 2010–2016. doi: 10.16420/j.issn.0513-353x.2011.10.023

[B67] Yamauchi A. Hosokawa A. Nagata H. Shimoda M. (2004). Triploid bridge and role of parthenogenesis in the evolution of autopolyploidy. Am. Nat. 164, 101–112. doi: 10.1086/421356 15266374

[B68] Yeh C. M. Chung K. Liang C. K. Tsai W. C. (2019). New insights into the symbiotic relationship between orchids and fungi. Appl. Sci. 3, 585. doi: 10.3390/app9030585

[B69] Yeung E. C. (2017). A perspective on orchid seed and protocorm development. Bot. Stud. 58, 33. doi: 10.1186/s40529-017-0188-4 28779349 PMC5544657

[B70] Yukawa T. Stern W. L. (2002). Comparative vegetative anatomy and systematics of *Cymbidium* (Cymbidieae: Orchidaceae). Bot. J. Linn. Soc 138, 383–419. doi: 10.1046/j.1095-8339.2002.00038.x

[B71] Zeng R. Z. Zhu J. Xu S. Y. Du G. H. Guo H. R. Chen J. J.. (2020). Unreduced male gamete formation in *Cymbidium* and its use for developing sexual polyploid cultivars. Front. Plant Sci. 11. doi: 10.3389/fpls.2020.00558 PMC7243674 32499802

[B74] Zhang X. Q. Cao Q. Z. Zhou P. Jia G. X. (2017). Meiotic chromosome behavior of the male-fertile allotriploid lily cultivar ‘Cocossa’. Plant Cell Rep. 36, 1641–1653. doi: 10.1007/s00299-017-2180-6 28741131

[B72] Zhang G. Q. Liu K. W. Li Z. Lohaus R. Hsiao Y. Niu S. C. (2017). The *Apostasia* genome and the evolution of orchids. Nature 549, 379–383. doi: 10.1038/nature23897 28902843 PMC7416622

[B73] Zhang W. X. Zhang G. Q. Zeng P. Zhang Y. Q. Hu H. Liu Z. J.. (2021). Genome sequence of *Apostasia ramifera* provides insights into the adaptive evolution in orchids. BMC Genomics 22, 536. doi: 10.1186/s12864-021-07852-3 34256691 PMC8278605

[B75] Zhang Y. X. Zhang G. Q. Zhang D. Y. Liu X. D. Xu X. Y. Sun W. H.. (2021). Chromosome-scale assembly of the *Dendrobium chrysotoxum* genome enhances the understanding of orchid evolution. Hortic. Res. 8, 183. doi: 10.1038/s41438-021-00621-z 34465765 PMC8408244

[B76] Zhou J. J. Zeng R. Z. Liu F. Yi M. S. Li Y. H. Zhang Z. S. (2009). Investigation on chromosome ploidy of the hybrids of *Phalaenopsis* polyploids (in Chinese). Acta Hortic. Sin. 10, 1491–1497. doi: 10.16420/j.issn.0513

[B77] Zhu J. Liu Y. Y. Zeng R. Z. Li Y. H. Guo H. R. Xie L.. (2014). Preliminarily study on formation and cytological mechanism of unreduced male gametes in different ploidy *Phalaenopsis* (in Chinese). Acta Hortic. Sin. 10, 2132–2138. doi: 10.16420/j.issn.0513-353x.2014.10.019

