# Supplementary figures and images for: Triploid cultivars of Cymbidium act as a bridge in the formation of polyploid plants
Source: Front Plant Sci. 2022 Oct 11;13:1029915. doi: 10.3389/fpls.2022.1029915 (PMC9853991; doi:10.3389/fpls.2022.1029915)

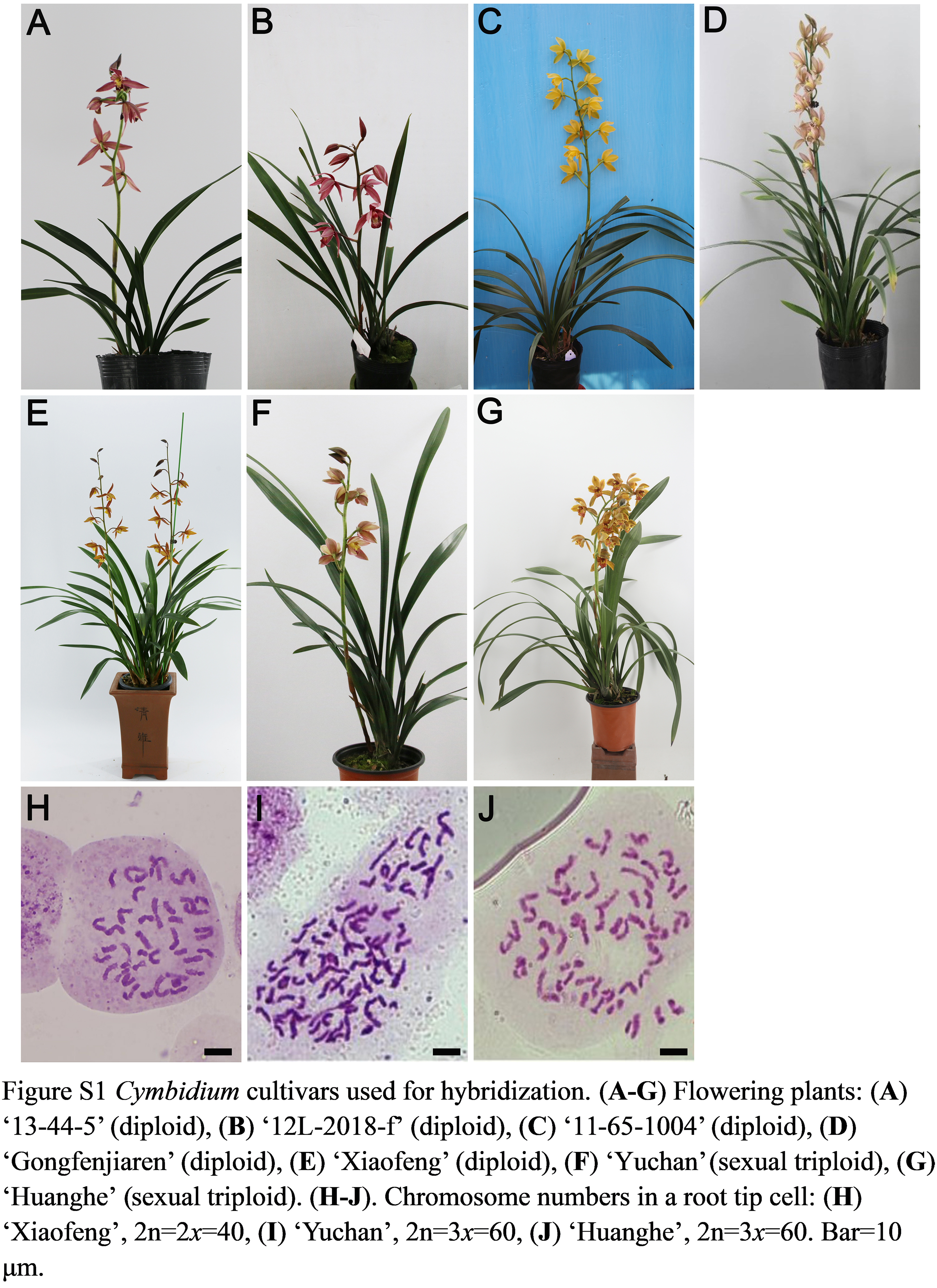

Supplement: Supplementary Figure 1 — Cymbidium cultivars used for hybridization. [file Image_1.jpeg]

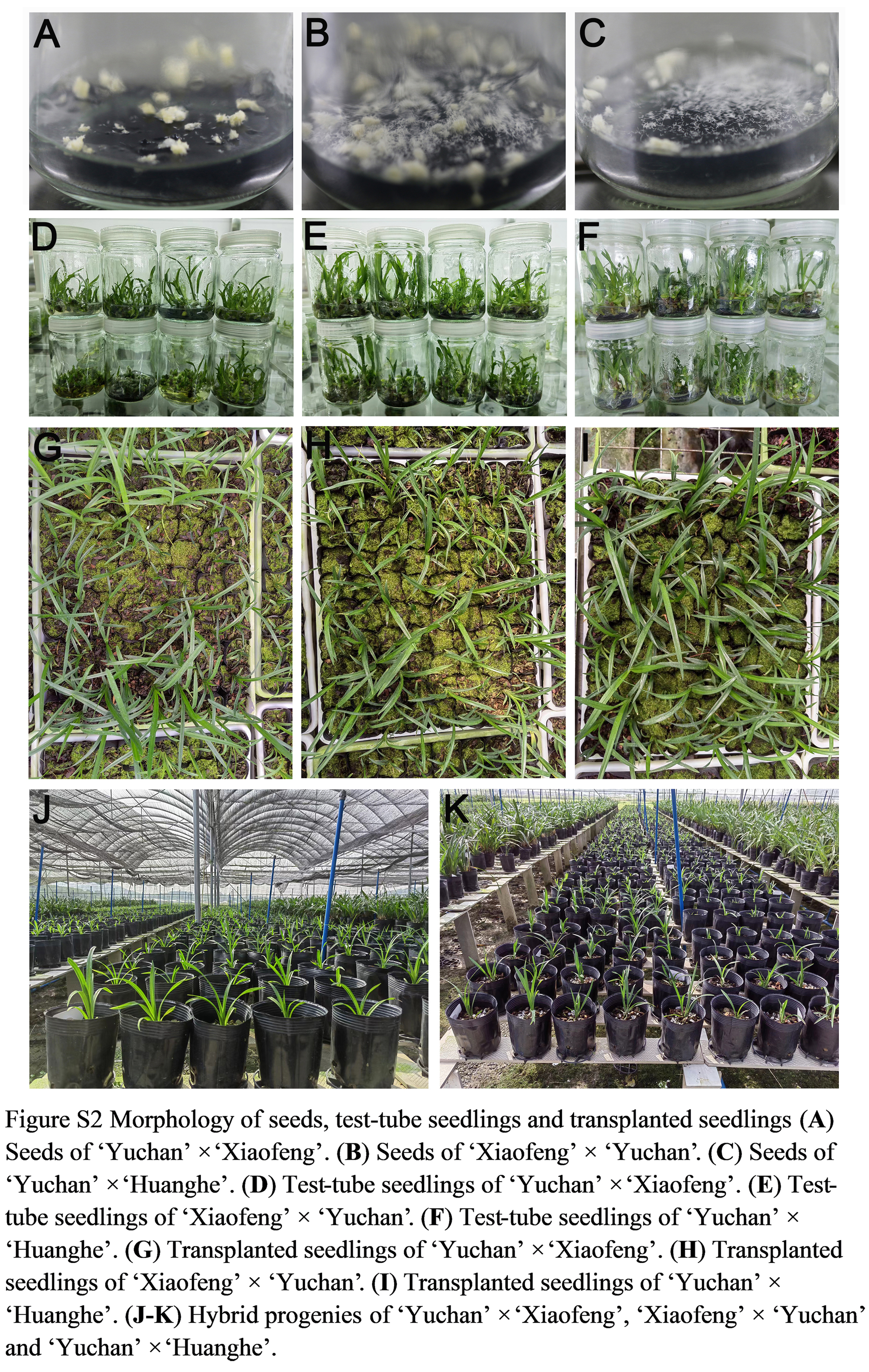

Supplement: Supplementary Figure 2 — Morphology of seeds, test-tube seedlings and transplanted seedlings. [file Image_2.jpeg]

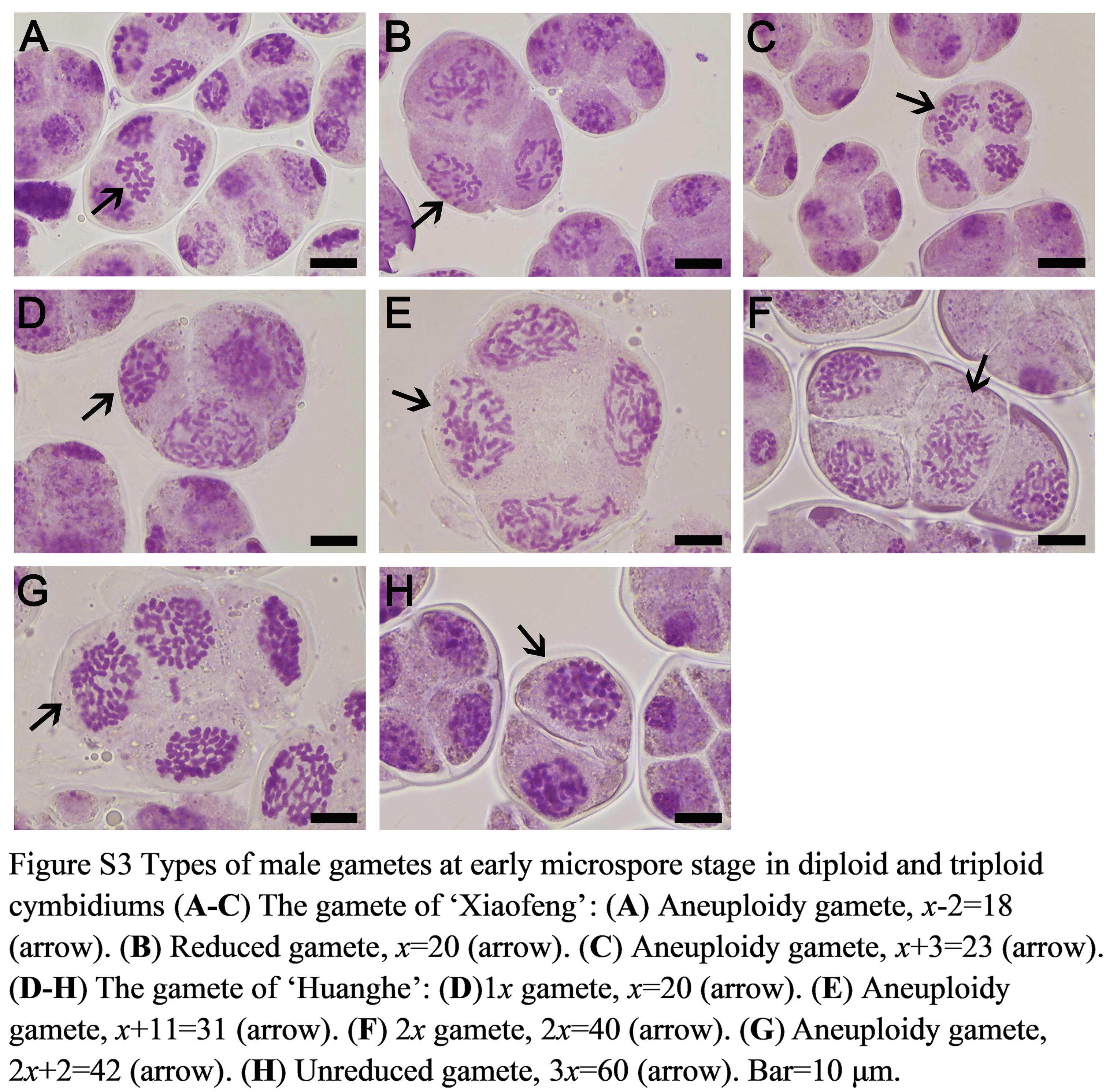

Supplement: Supplementary Figure 3 — Types of male gametes at early microspore stage in diploid and triploid Cymbidium. [file Image_3.jpeg]

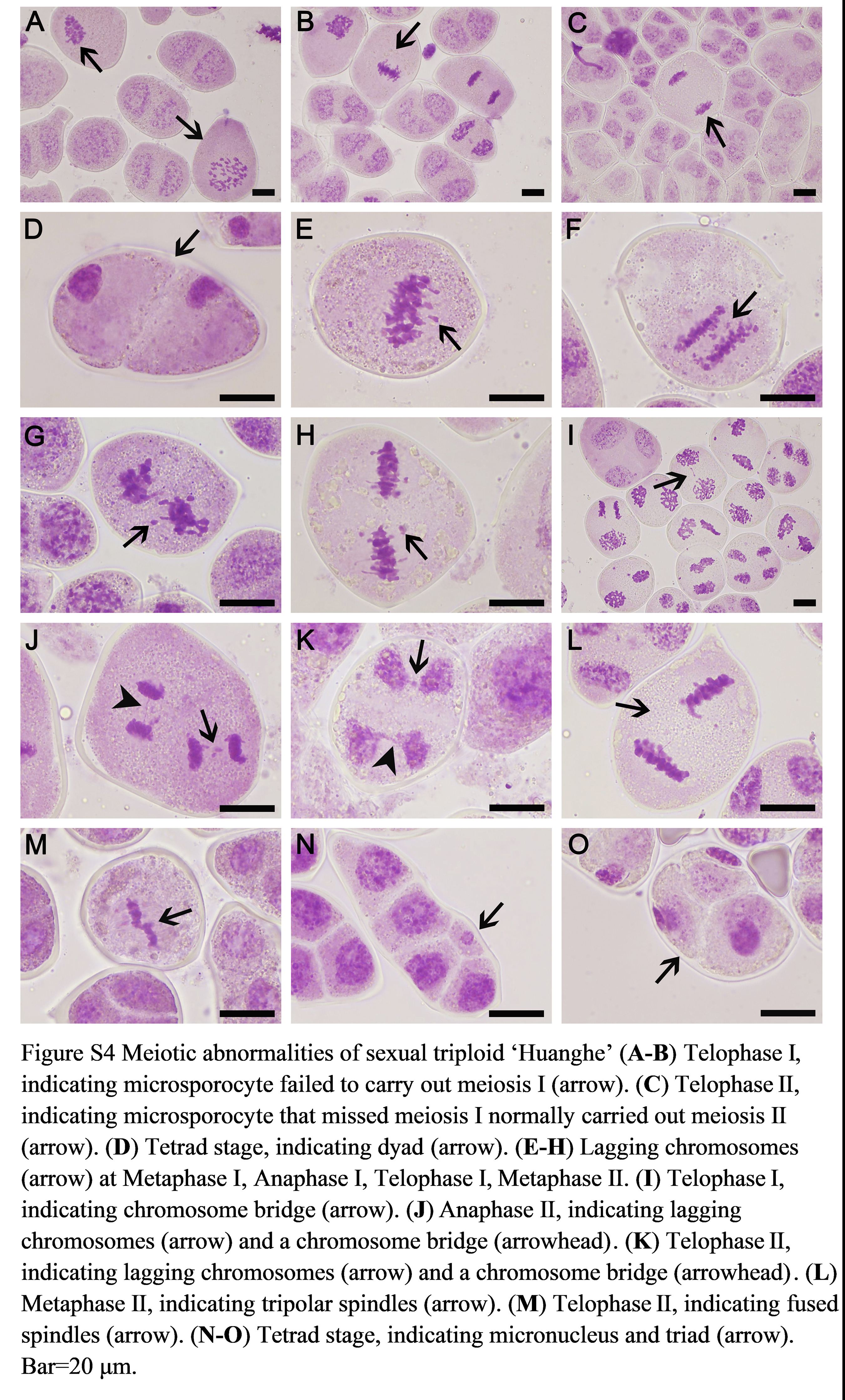

Supplement: Supplementary Figure 4 — Meiotic abnormalities of sexual triploid ‘Huanghe’. [file Image_4.jpeg]

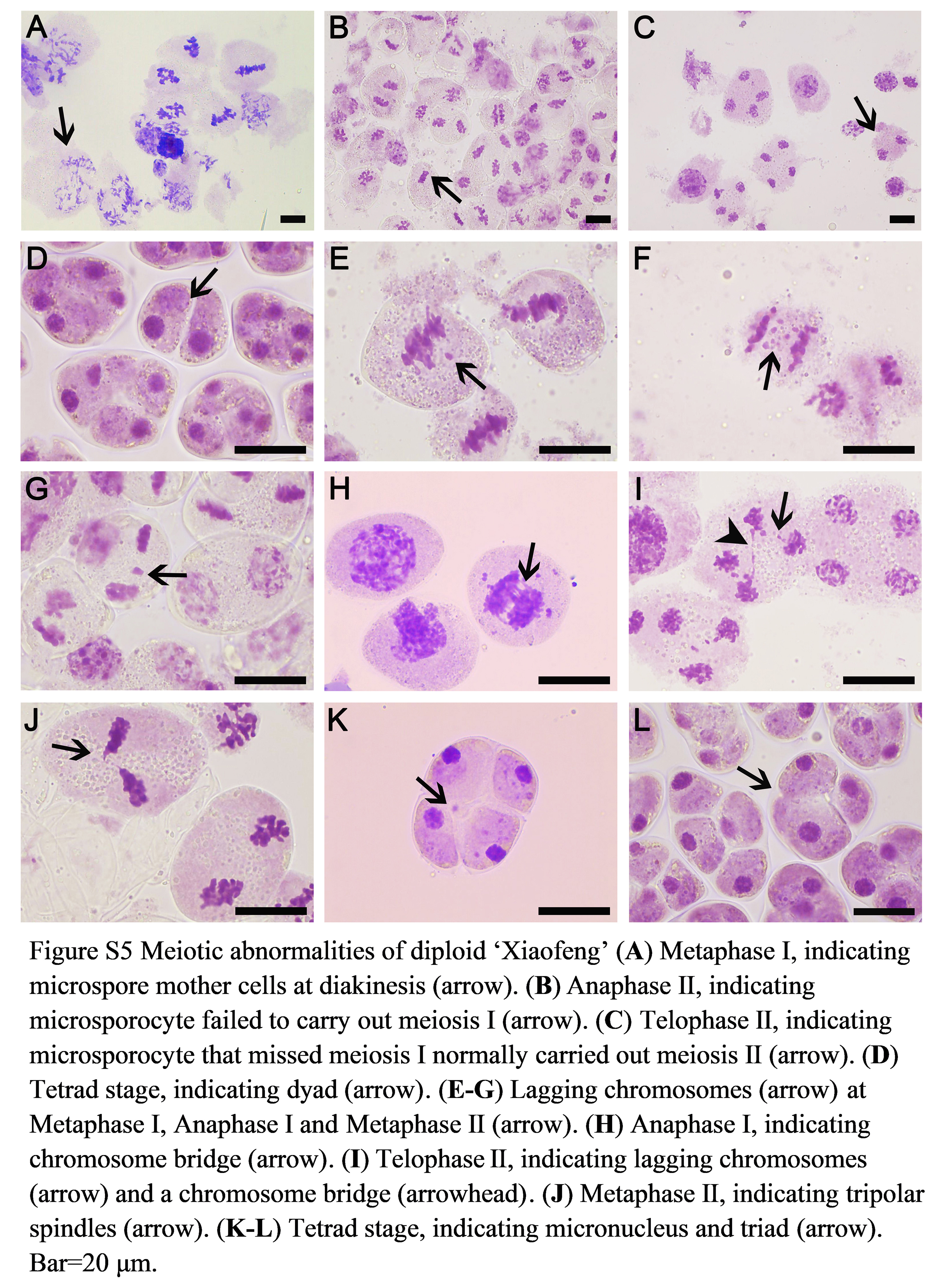

Supplement: Supplementary Figure 5 — Meiotic abnormalities of diploid ‘Xiaofeng’. [file Image_5.jpeg]
